# What outcomes in community mental health research are important to caregivers of people with schizophrenia? An exploratory qualitative analysis of an online survey

**DOI:** 10.1002/npr2.12295

**Published:** 2022-10-10

**Authors:** Naonori Yasuma, Takuma Shiozawa, Makoto Ogawa, Makiko Abe, Momoka Igarashi, Takayuki Kawaguchi, Sayaka Sato, Daisuke Nishi, Norito Kawakami, Sosei Yamaguchi, Chiyo Fujii

**Affiliations:** ^1^ Department of Community Mental Health and Law, National Institute of Mental Health National Center of Neurology and Psychiatry Kodaira Tokyo Japan; ^2^ Ageonomori Clinic Ageo Saitama Japan; ^3^ Department of Mental Health, Graduate School of Medicine The University of Tokyo Bunkyo‐ku Tokyo Japan; ^4^ Department of Digital Mental Health, Graduate School of Medicine The University of Tokyo Bunkyo‐ku Tokyo Japan

**Keywords:** caregivers, clinical, community mental health, outcomes, schizophrenia

## Abstract

**Aims:**

This study investigated outcomes in community mental health research that were important to caregivers of people with schizophrenia.

**Methods:**

Using an online survey conducted from August 1 to 31, 2020, data were collected from caregivers belonging to the LINE Schizophrenia Family Association. Caregivers identified outcomes important in community mental health research. Two researchers categorized caregivers' statements into research outcomes.

**Results:**

A total of 132 caregivers completed the online selfreported questionnaire, and 296 caregiver statements were identified. Qualitative analysis identified 17 outcome categories. The caregivers tended to value having more free time, maintaining an appropriate relationship with people with schizophrenia, and being able to cope with their symptoms.

**Conclusions:**

This exploratory study newly demonstrates the outcomes that caregivers of people with schizophrenia consider important in community mental health research. The findings may be useful in selecting outcomes for future studies of caregivers.

## 1 INTRODUCTION

Outcome selection and participant involvement are important in modern clinical and research work [1]. This appears to apply to studies of caregivers of people with mental illness since they have specific needs and require effective support [2]. Importantly, caregivers appear to have different concerns from people with mental illness. For example, they tend to bear significant physical, mental, and economic burdens as a result of caring for those with mental illness [3]. In short, we need to identify proper research outcomes for caregivers themselves.

A previous systematic review reported outcomes from the past three decades that were considered important to caregivers [4]. These outcomes were mainly divided into three dimensions, namely negative aspects of caregiving (e.g., strain, stress, and worrying), positive aspects of caregiving (e.g., personal growth, strength, support, rewards, and satisfaction), and caregivers' perceptions and attitudes toward people with mental illness (e.g., insight, stigma, and efficacy). Most studies primarily assessed the negative aspects of caregiving, while positive aspects of caregiving have received less attention [4]. Other systematic reviews also pointed out that few studies have focused on caregivers' recovery and well‐being [5, 6]. Therefore, further research is expected to explore outcomes meaningful to caregivers.

Despite an increasing emphasis on outcomes important to caregivers, the outcomes in most individual studies may still be determined by researchers rather than caregivers [7]. Focusing on caregivers' experiences, needs, and interests in the outcome selection process appears to be important for identifying meaningful goals and sharing research findings. In fact, people with mental illness and researchers often recognized different needs and goals for treatment [8], and this may also be true for caregivers and researchers. In addition, caregiver involvement has been accelerated by a recent trend toward patient public involvement (PPI), or coproduction [9, 10]. Therefore, exploring outcomes that caregivers consider important can be essential to promote future studies that meet their needs. Although several systematic reviews have summarized experiences or views of caregivers regarding caring for people with mental illness [5, 11, 12, 13, 14], few studies have directly asked caregivers about important outcomes in research, particularly in community‐based mental health research settings. This study preliminarily investigated what outcomes are deemed by caregivers of people with mental illness to be important for community‐based mental health research.

## 2 METHODS

### 2.1 Study design and participants

An online, cross‐sectional survey was conducted from August 1 to 31, 2020. Participants were recruited from the LINE Family Association of Schizophrenia, which had 238 registered members as of August 1, 2020. The association offers a platform for information exchange and peer support for caregivers of people with schizophrenia using the “LINE” online software. Eligibility criteria were as follows: (a) caregiver of one or more people with schizophrenia and (b) age over 20 years. For example, a caregiver might care for a family member with schizophrenia and look after his or her daily needs. Respondents were invited to participate on a voluntary basis and provided fully informed consent through the internet. The study protocol was approved by the Research Ethics Committee of the National Center of Neurology and Psychiatry (No. A2020‐036).

### 2.2 Data collection

We collected sociodemographic data of participating caregivers, such as age, gender, education, and annual household income. We also assessed care experiences, such as relationship with people with schizophrenia, time spent providing care, and caregiver burden as evaluated using the Japanese version of the Zarit burden interview, short version (J‐ZBI‐8) [15]. The data on family members with schizophrenia were obtained from their caregivers, including age, gender, duration of illness, and lifetime number of hospitalizations. We defined the following open‐ended question to capture outcomes in community mental health research that are important to caregivers of people with schizophrenia: “What benefits do you (i.e., caregivers) wish to gain from support services? Please enter up to three ideas.” We created the questions through discussion with caregivers to enable them to easily understand the term “outcome” which was unfamiliar to them. We also worked with caregivers to modify the online survey screens in order for participants to answer each question easily. We held an online conference for the participating caregivers to share the results of our research on March 20, 2021. We then confirmed their supportive comments on the results and no requests to change the results.

### 2.3 Analyses

Two researchers (NY and TS) independently extracted outcomes based on participants' survey responses and then organized word fluctuation of the responses. Next, related responses were collected and categorized through joint analysis and thorough discussion between the two researchers. Each category whose meaning was ultimately unclear was excluded. For example, responses that discussed the outcomes of people with schizophrenia, rather than those of their caregivers, were excluded. When disagreement occurred between the two researchers, decisions were made based on the advice of SY and MO. The category names were iteratively reviewed to ensure that they reflected the literal meanings of the responses. We shared the analysis results with several caregivers who participated in the survey to obtain their feedback. We then incorporated it into our analysis. A co‐author (TK) not involved in the analysis fit 30 randomly selected outcome statements into 17 categories. The agreement rate was calculated using Krippendorff's alpha [16], and the criterion for good reliability was set at 0.8 [16]. Finally, the number of responses corresponding to each category was calculated. The categories are presented in Table 2.

## 3 RESULTS

**FIGURE 1:**
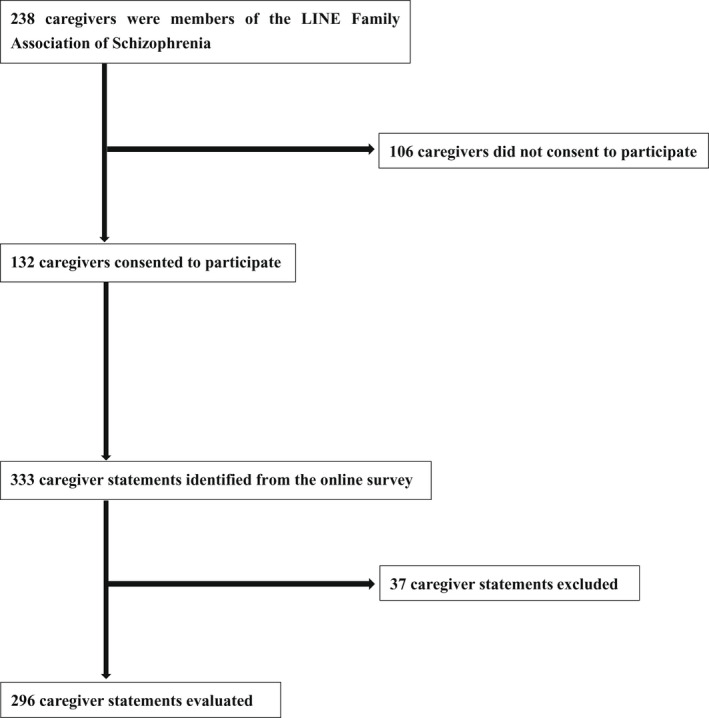
Study flowchart

A study flowchart is shown in Figure 1. A total of 132 caregivers completed the online selfreported questionnaire and 296 caregiver statements were identified. The categorization agreement rate was 0.82. Caregiver characteristics are presented in Table 1. Most caregivers were middle‐aged mothers with a care duration of less than 5 years. The analysis resulted in 17 outcome categories in which the participating caregivers were interested regarding community mental health research. Table 2 shows a ranking of important outcome categories. The category with the highest number of responses concerned the desire to have more free time. This was followed by categories related to maintaining an appropriate relational distance with people with schizophrenia and the ability to cope with these people's symptoms. Eight of the top 10 items involved caregivers’ personal lives, care burden, and relationships.

**TABLE 1:** Demographic characteristics of caregivers and people with schizophrenia (N = 132)

	n (%) or Mean (SD)
Caregivers	
Age	52.7 (7.9)
Gender	
Male	11 (8.3)
Education	
Junior high school	2 (1.5)
High school	21 (15.9)
Some college	54 (40.9)
University or higher	55 (41.7)
Annual household income	
<2.5 million yen	19 (14.4)
<5 million yen	25 (18.9)
<7.5 million yen	29 (22.0)
≥7.5 million yen	48 (36.4)
Unknown	11 (8.3)
Relationship to person with schizophrenia	
Parent	111 (84.1)
Spouse	9 (6.8)
Sibling	6 (4.5)
Child	6 (4.5)
Care duration	
≤5 years	82 (62.1)
≤10 years	24 (18.2)
≤15 years	16 (12.1)
≤20 years	3 (2.3)
≥21 years	7 (5.3)
Japanese version of the Zarit burden interview, short version (J‐ZBI‐8)	15.4 (7.9)
People with schizophrenia	
Age	27.7 (11.6)
Gender	
Male	63 (47.7)
Duration of illness	
≤5 years	82 (62.1)
≤10 years	20 (15.2)
≤15 years	18 (13.6)
≤20 years	3 (2.3)
≥21 years	9 (6.8)
Lifetime number of hospitalizations	1.6 (1.6)

**TABLE 2:** Ranking of outcomes considered by caregivers of people with schizophrenia to be important in community mental health research

Ranking	Item	Description	Number of statements
1	Having more free time	Caregivers want more free time for themselves	48
2	Maintaining an appropriate relationship with people with schizophrenia	Caregivers want to maintain an appropriate relationship with those they care for	47
3	Coping with symptoms of people with schizophrenia	Caregivers want to cope well with the symptoms or medical conditions of those they care for	36
4	Decreasing financial burden of patients with schizophrenia	Caregivers do not want to carry the financial burden of those they care for	28
5	Decreasing daily care	Caregivers want to reduce the amount of care they must provide	27
6	Decreasing psychological burden	Caregivers want to feel less of a psychological burden related to their caregiving duties	22
7	Living their own life	Caregivers want to live their own lives without being influenced by those they care for	19
8	Living separately from people with schizophrenia	Caregivers want to live separately from those they care for.	19
9	Working	Caregivers want to work without worrying about those they care for	15
10	Stigma	Caregivers want to live in the community without worrying about stigma related to schizophrenia	15
11	Obtaining information about schizophrenia	Caregivers want to obtain knowledge and information about schizophrenia	8
12	Using mental health services	Caregivers want to use mental health and welfare services to ease their care burden.	3
13	Connecting with other caregivers	Caregivers want to be connected with others in the same situation	3
14	Living together with people with schizophrenia	Caregivers want to be able to live together with those they care for	2
15	Disseminating information about schizophrenia	Caregivers want to disseminate information about schizophrenia to society	2
16	Being healthy	Caregivers want to be healthy	1
17	Having someone to complain to	Caregivers want to have someone to complain to	1
	Excluded		37
	Total		333

## 4 DISCUSSION

This study suggested outcomes that caregivers of people with schizophrenia identified as important in community mental health research. Caregivers tended to value having more free time, maintaining an appropriate relational distance with people with schizophrenia, and the ability to cope with these people's symptoms.

Caregiver burden is considered as the main outcome identified by previous studies, and positive aspects of caregiving, namely caregiver recovery and well‐being, have been recognized less frequently [4, 5, 6]. Since caregiver burden captures the degree of multifaceted burden that caregivers experience from caring for family members and partners over time [17], the results of this study may not cover all aspects of caregiver burden. However, the overall trend toward family burden as an important outcome appears to be similar to previous studies.

The reason for these results may be related to weaknesses in the community‐based mental health system in Japan. For example, 90% of health professional work in inpatient settings in Japan, and the number of psychiatric beds is still the highest across the world [18]. In addition, for almost 100 years until 2014, Japanese family members statutorily were obligated to provide care for people with mental illness. A study suggests feelings of care burden of family in Japan is higher than those in Korea [19]. Although there has been a gradual increase in services for people with mental illness, the community‐based mental health system is mainly designed to provide facility‐based services in Japan [20]. In other words, community services are inadequate, particularly for those with severe mental illness or those in the acute phase of recovery who have difficulty going out. Consequently, caregivers often play a major role in supporting their daily lives. The common outcomes identified in this study may reflect this situation. Specifically, they may indicate that caregivers need a community‐based mental health system in which they do not bear so much responsibility for caring for people with mental illness.

The strength of this study is that we worked with caregivers in the development stage of the question and online survey screens based on the concepts of PPI [9, 10], although the PPI could not be performed in all the stages of this study. This could lead to future interventional studies whose primary outcomes are based on what caregivers consider to be highly important, as well as additional, implementable, evidence‐based practices for caregivers. On the contrary, this study had several limitations. First, the study was not conducted by the family caregivers themselves. While we obtained the feedback from the caregivers, it was still impossible to avoid the researcher's interpretation of the analysis. Second, the amount of interview data may be inadequate. Although this study included over 100 caregivers and obtained a wide variety of statements, more in‐depth interviews using semistructured methods might have yielded more information than this study. Third, outcomes were ranked by numbers of responses, but this approach may be inadequate to determine which outcomes are most important. Fourth, we did not consider covariates related to caregiver characteristics and circumstances because we could not link participants’ statements with their personal characteristics in this online survey. Further research employing a more rigorous qualitative design is needed to confirm the findings of this study and to assess covariate influence.

Despite these limitations, this exploratory study suggested outcomes that caregivers of people with schizophrenia considered important in community mental health research. The findings may be useful in selecting outcomes for future studies of caregivers.

## AUTHOR CONTRIBUTIONS

NY wrote the first draft of the manuscript, and other authors critically revised the manuscript. All authors approved the final manuscript.

## CONFLICT OF INTEREST

The authors declare no conflict of interest.

## ETHICAL APPROVAL

The study was approved by the Research Ethics Committee of the National Center of Neurology and Psychiatry (No. A2020‐036).

## INFORMED CONSENT

Informed consent was obtained from all subjects.

## Data Availability

Not all data are freely accessible because no informed consent was given by the participating agencies for open data sharing. However, the data are available from the corresponding author on reasonable request, following approval by the Research Ethics Committee at the National Center of Neurology and Psychiatry.
